# Elastase and Tryptase Govern TNFα-Mediated Production of Active Chemerin by Adipocytes

**DOI:** 10.1371/journal.pone.0051072

**Published:** 2012-12-05

**Authors:** Sebastian D. Parlee, Jenna O. McNeil, Shanmugam Muruganandan, Christopher J. Sinal, Kerry B. Goralski

**Affiliations:** 1 College of Pharmacy, Dalhousie University, Halifax, Nova Scotia, Canada; 2 Department of Pharmacology, Dalhousie University, Halifax, Nova Scotia, Canada; University of Bern, Switzerland

## Abstract

Chemerin is a leukocyte chemoattractant and adipokine with important immune and metabolic roles. Chemerin, secreted in an inactive form prochemerin, undergoes C-terminal proteolytic cleavage to generate active chemerin, a ligand for the chemokine-like receptor-1 (CMKLR1). We previously identified that adipocytes secrete and activate chemerin. Following treatment with the obesity-associated inflammatory mediator TNFα, unknown adipocyte mechanisms are altered resulting in an increased ratio of active to total chemerin production. Based on these findings we hypothesized adipocytes produce proteases capable of modifying chemerin and its ability to activate CMKRL1. 3T3-L1 adipocytes expressed mRNA of immunocyte and fibrinolytic proteases known to activate chemerin *in vitro*. Following treatment with a general protease inhibitor cocktail (PIC), the TNFα-stimulated increase in apparent active chemerin concentration in adipocyte media was amplified 10-fold, as measured by CMKLR1 activation. When the components of the PIC were investigated individually, aprotinin, a serine protease inhibitor, blocked 90% of the TNFα-associated increase in active chemerin. The serine proteases, elastase and tryptase were elevated in adipocyte media following treatment with TNFα and their targeted neutralization recapitulated the aprotinin-mediated effects. In contrast, bestatin, an aminopeptidase inhibitor, further elevated the TNFα-associated increase in active chemerin. Our results support that adipocytes regulate chemerin by serine protease-mediated activation pathways and aminopeptidase deactivation pathways. Following TNFα treatment, increased elastase and tryptase modify the balance between activation and deactivation, elevating active chemerin concentration in adipocyte media and subsequent CMKLR1 activation.

## Introduction

Chemerin is an adipocyte-secreted protein (adipokine) that regulates adipogenesis, metabolism and inflammation through activation of the chemokine-like receptor 1 (CMKLR1) [1]–[9]. Human serum or plasma concentrations of total chemerin (measure of inactive and active chemerin forms) correlates with markers of inflammation, are elevated in individuals with obesity, coronary artery disease, insulin-resistance, early onset and long-term type 2 diabetes and hypertension [10]–[24]. The collective findings indicate that chemerin concentrations are modifiable in obesity and may contribute to human diseases that are associated with obesity and have an inflammatory component [11], [12], [25], [26]. However, continued efforts to clarify the factors that regulate adipocyte production of active chemerin are required to understand why chemerin concentrations become elevated in obesity and to better assess the functional impact of chemerin/CMKLR1 signalling in obesity related disease progression.

The currently held belief surrounding chemerin production and activity proposes that chemerin is secreted as a 163 amino acid inactive protein prochemerin that subsequently undergoes proteolysis at sites of tissue injury to form active chemerin. Six separate full-length human chemerin products have been identified *in vitro* which differ by both their C-terminal amino acid and ability to activate CMKLR1. These include; chemerin_158_, *_−_*_157_, *_−_*_156_, *_−_*_155, *−*154_ and *_−_*_152_, with subscript numbers referring to the terminal amino acid position of the processed protein. The serine proteases plasmin and mast cell tryptase cleave prochemerin into chemerin_158_, a weak activator of CMKLR1 [27]. Sequential removal of the c-terminal lysine from chemerin_158_ by plasma carboxypeptidase N or B forms chemerin_157_, the chemerin product with the highest known activity at CMKLR1 [28], [29]. In contrast, neutrophil elastase and cathepsin G, K and L cleave 6 or 7 C-terminal acids from prochemerin to produce the potent CMKLR1 agonists chemerin_157_ and chemerin_156_, respectively [27], [30], [31]. Proteolytic processing is also responsible for deactivation or degradation of chemerin. Mast cell chymase or angiotensin converting enzyme converts chemerin_157_ into inactive chemerin_154_ or low active chemerin_152_ [32], [33]. Fibrinolytic enzymes including tissue plasminogen activator (tPA), plasminogen activator urokinase (uPA) and plasminogen also activate prochemerin although the resulting chemerin products are unknown [9]. Synthetic peptides corresponding to the final C-terminal amino acids of chemerin can activate CMKLR1 *in vitro* with low nanomolar potency [29] and a peptide corresponding to amino acids 140–154 of the chemerin C-terminus has been reported to trigger anti-inflammatory processes in cultured macrophages and zymozan-induced peritonitis in mice via CMKLR1 signalling [34]. Thus a second possibility is proteolytic enzymes split the C-terminal ending from full length chemerin, resulting in the formation small active peptides. Such C-terminal peptides have not been identified as endogenous products *in vitro* or *in vivo*; thus, their physiological relevance is uncertain. Although studies investigating chemerin processing have focused on the human variant, mouse prochemerin has similar predicted proteolytic cleavage sites based on the sequence homology with the C-terminus of human chemerin [35], [36], [37]. Collectively, the *in vitro* results support that CMKLR1-mediated chemerin functions are dependent upon local proteolytic mechanisms capable of activating and deactivating chemerin. These processes could be especially important in obese white adipose tissue, which secretes elevated amounts of chemerin [5].

In our previous study we identified that adipocytes produce active chemerin under basal conditions as measured by the ability of conditioned adipocyte media to activate CMKLR1 in a cell-based bioassay. Following treatment with an obesity-associated inflammatory mediator TNFα, the ratio active to total immunodetectable chemerin in adipocyte media increased [38]. These findings support the novel concept that adipocytes contain the machinery needed to secrete and process prochemerin to its active products. Thus, adipocytes could be an important modifiable source of local and systemic concentrations of active chemerin. Currently, however, no documented information is available concerning the proteolytic mechanisms that regulate chemerin activation by adipocytes.

The overall study object was to define the proteolytic mechanisms that control chemerin activation under basal conditions and following treatment with TNFα in 3T3-L1 cells and primary bone mesenchymal stromal cells (BMSCs), two well-established adipogenic models. Taken together, the findings from our study established that adipocytes regulate the production of active chemerin through a balance of protease-mediated activation and deactivation pathways. Following treatment with TNFα, elevated concentrations of the serine proteases elastase and tryptase modify the balance between activation and deactivation, resulting in a net elevation in active chemerin in adipocyte media as measured by CMKLR1 activation.

## Methods

### 3T3-L1 and BMSC Adipocyte Cell Culture

3T3-L1 preadipocytes were obtained from the American Tissue Culture Collection (Manassas, VA) and were grown and differentiated according to our published protocols [2]. Primary BMSC-derived adipocytes were isolated and cultured from 8-wk-old C57BL6/J mice bred in-house using established methods [3]. The Dalhousie University Committee on Laboratory Animals approved the experimental procedures involving mice and the procedures were carried out in accordance with the regulations of the Canadian Council on Animal Care. All adipocyte media was phenol red free. For the protease mRNA expression experiments, cell lysates from 3T3-L1 cells grown on 12-well plates were harvested at the preadipocyte (day 0), adipocyte differentiation (days 3 and 5) and mature adipocyte (days 8 and 13) stages and the subsequent total cellular RNA was isolated. For the protease inhibitor experiments, 13-day (3T3-L1) or 26-day (BMSC) adipocytes grown on 12-well plates were rinsed once with 1 mL of PBS and then treated with 20 ng mL^−1^ recombinant mouse TNFα (R&D Systems, Minneapolis, MN) or 0.1% BSA in PBS (0.1% BSA/PBS) vehicle in combination with a 1∶200 diluted protease inhibitor cocktail, PIC (Catalogue #P1860, Sigma Aldrich, Oakville, ON) containing a proprietary combination of serine (aprotinin), cysteine (E–64), aminopeptidase (bestatin) and aspartyl (pepstatin A) protease inhibitors or individually with aprotinin (0.1–30 µM), E-64 (1–100 µM), bestatin (2–60 nM) in 500 µL of serum-free DMEM for a period of 24 h. In a similar fashion, additional protease inhibitor experiments were performed with the pan carboxypeptidase inhibitor, potato tuber carboxypeptidase inhibitor (1–20 nM). Each experiment included the appropriate vehicle control treatments, which were H_2_O containing 0.9% Benzyl Alcohol and 0.9% NaCl (pH 5.7–6.2) for aprotinin, H_2_O for E-64, bestatin and the carboxypeptidase inhibitor and DMSO for the PIC. After the 24 h incubation, the conditioned adipocyte media or whole cell lysate was collected and the corresponding cellular RNA was isolated. Media and RNA samples were stored at −80°C until western blots, chemerin bioassays and gene expression analyses were performed.

### RNA Isolation and Quantitative PCR

Total cellular RNA was isolated using the Bio-Rad aurum total RNA mini kit (Bio-Rad, Mississauga, ON) according to the manufacturer’s instructions and 0.5 µg was reverse transcribed using Superscript III Reverse Transcriptase (Life Technologies Inc., Burlington, ON). 0.5 µL of the cDNA product was amplified by quantitative PCR (QPCR) using 125 nM of gene specific primers in a total volume of 20 µL with iTaq SYBR green supermix with ROX QPCR master mix (Bio-Rad) using the StepOnePlus Real-Time PCR system (Life Technologies Inc.). Relative gene expression normalized to *Cyclophilin A* expression was calculated using the cycle threshold (ΔΔC_T_) method [39]. Agarose gel electrophoresis of the QPCR products confirmed that each set of QPCR primers produced a single band of the correct molecular size. The forward and reverse QPCR primer sequences and product sizes are identified in Table 1.

**Table 1:** Quantitative-PCR primer gene identification, sequences and product sizes.

Mouse Gene	Identification	Sequence	Product Size (bp)
*neutrophil elastase*	NM_015779.2	TGTGAACGGCCTAAATTTCC	186
		ACGTTGGCGTTAATGGTAGC	
*mast cell tryptase*	NM_031187	GAGACCTTCCCCTCAGGAAC	200
		ATGTCCTTCATTCCCAGCAC	
*tPA*	NM_008872	GCTGAGTGCATCAACTGGAA	243
		GCCACGGTAAGTCACACCTT	
*uPA*	NM_008873	AGTGTGGCCAGAAGGCTCTA	279
		GCTGCTCCACCTCAAACTTC	
*cathepsin K*	NM_007802.3	CAGCTTCCCCAAGATGTGAT	165
		AGCACCAACGAGAGGAGAAA	
*angiotensin converting enzyme*	NM_001130513	CAGTGTCTACCCCCAAGCAT	101
		GTGAGGGCCATCTTCATTA	
*cyclophilin*	X52803.1	GAGCTGTTTGCAGACAAAGTTC	134
		CCCTGGCACATGAATCCTGG	
*Carboxypeptidase N*	NM_030703.2	ACATCCTGCCTTCCATGAAC	279
		CGTGCATATTGGCTGAGAGA	
*Carboxypeptidase B*	NM_019775	AGAAGCAAAAGCAAGGACCA	249
		TGCACAAGTGGGTTTGATGT	

### Quantification of Active Chemerin in Adipocyte Media Using the CMKLR1 “Tango” Bioassay

The CMKLR1 “Tango” bioassay is a cell-based assay that specifically and quantitatively measures CMKLR1 activation by active chemerin. By comparing mouse CMKLR1 activation by media samples to known concentrations of recombinant active mouse chemerin_156_ (R&D Systems) we are able to determine the apparent sample concentration of active chemerin expressed as mouse chemerin_156_ equivalents. A detailed description of the assay procedures has been described previously [38], [40]. To exclude potential non-specific effects of serine, cysteine and aminopeptidase inhibition on the CMKLR1 bioassay, a vehicle control, 30 µM aprotinin, 60 nM bestatin or 100 µM E-64 in 120 µL of serum free DMEM was spiked with 0, 1, 3 or 10 nM recombinant *m-*chemerin_156_ and subsequently analyzed by the CMKLR1 bioassay. The specific activation of the CMKLR1 bioassay by chemerin was determined by incubating the 24 h conditioned media from 3T3-L1 or BMSC-derived adipocytes for 1 h with 10 µg mL^−1^ of goat anti-mouse chemerin neutralization antibody (R&D Systems) or 10 µg mL^−1^ of goat IgG control antibody (Life Technologies Inc.) prior to analyzing the samples in the CMKLR1 bioassay. To identify whether the activation of CMKLR1 was due to the full length (16 kDa) chemerin protein and/or smaller (<3 kDa) proteolytic peptide fragments of chemerin, 240 µL of 24 h conditioned media from 3T3-L1 adipocytes was centrifuged at 4°C for 20 minutes at 8000×g through a 10 kDa size exclusion spin column (Pall Corporation, Ann Arbor, MI) prior to analysis by the CMKLR1 bioassay. The 240 µL filtered fraction (molecules <10 kDa) was collected as is and, the residue remaining in the column filter (molecules >10 kDa) was resuspended in 240 µL of DMEM/0.1% BSA.

### Neutralization of Neutrophil Elastase and Mast Cell Tryptase

The effects of neutrophil elastase and mast cell tryptase on active chemerin following treatment with TNFα or a vehicle control was investigated using neutralizing antibodies directed towards the C-terminus and internal region of mouse elastase and tryptase, respectively. 3T3-L1 adipocytes were preincubated for 1 h with 10 µg mL^−1^ goat IgG (R&D Systems), 5 µg mL^−1^ goat-anti-mouse neutrophil elastase (Catalogue #sc-9521, Santa Cruz Biotechnology Inc., Santa Cruz, CA), 5 µg mL^−1^ goat-anti-mouse mast cell tryptase (Catalogue #sc-32474, Santa Cruz Biotechnology Inc.) or a combination of 5 µg mL^−1^ of anti-elastase and 5 µg mL^−1^ anti-tryptase antibodies in 250 µL of serum free DMEM. Then 250 µL of serum free DMEM containing 40 ng mL^−1^ TNFα (final concentration 20 ng mL^−1^) or 0.1% PBS/BSA control was added to each well of adipocytes and the incubated for 24 h prior to harvesting the media for analysis by the CMKLR1 bioassay.

### SDS-Page Western Blotting

20 µL of 24 h conditioned adipocyte media or 7.5 ng of recombinant mouse chemerin_156_ was added to 6× sodium dodecyl sulfate loading buffer containing β-mercaptoethanol and incubated at 95°C for 5 min. The resulting solution was separated on a 15% polyacrylamide gel and transferred overnight to a nitrocellulose membrane. The nitrocellulose was subsequently washed with TBS for 5 min and incubated in 10 mL of Odyssey blocking buffer (LI-COR Biosciences, Lincoln, NE) containing 0.1% tween-20 (called western buffer) for 1 h. Once blocking was complete, the nitrocellulose was placed overnight in western buffer containing either a 1∶200 dilution of goat anti-mouse chemerin (R&D Systems), neutrophil elastase (M-18) or mast cell tryptase (G-12) (Santa Cruz, Biotechnology Inc.) or a 1∶50 dilution of goat anti-mouse tPA (C-16) or uPA (M-20) (Santa Cruz, Biotechnology Inc.). The following day the blot was washed 4 times for 5 min in TBS-T and incubated for 1 h in a 1∶5000 dilution of a 680 or 800 nm infrared fluorophore-conjugated polyclonal donkey anti-goat IgG (LI-COR Biosciences). The nitrocellulose was finally washed 4 times for 5 min in TBS-T and switched into TBS prior to scanning at 700 or 800 nm at a resolution of 84 µM using a LI-COR Odyssey infrared scanner at an intensity of 5 (chemerin, elastase and tryptase) or 6 (tPA and uPA). LI-COR Odyssey imaging software was used to quantify the relative band intensities on the chemerin western blots.

### Statistical Analysis

All data are expressed as mean ± s.e.m of 3 samples, and are representative of at least 2 independent experiments. Statistical analysis was performed using GraphPad Prism. A two-way analysis of variance (ANOVA) was used for comparing proteolytic inhibitor-dependent effects on the basal and TNFα-stimulated apparent active chemerin concentration in adipocyte media. A one-way ANOVA was used for multiple comparison procedures with one independent variable. A Tukey’s or Bonferroni test was used for *post-hoc* analysis of the significant ANOVA. A difference in mean values between groups was considered to be significant when P ≤ 0.05.

## Results

### Activation of CMKLR1 by Adipocyte Media is Chemerin-specific

**Figure 1:**
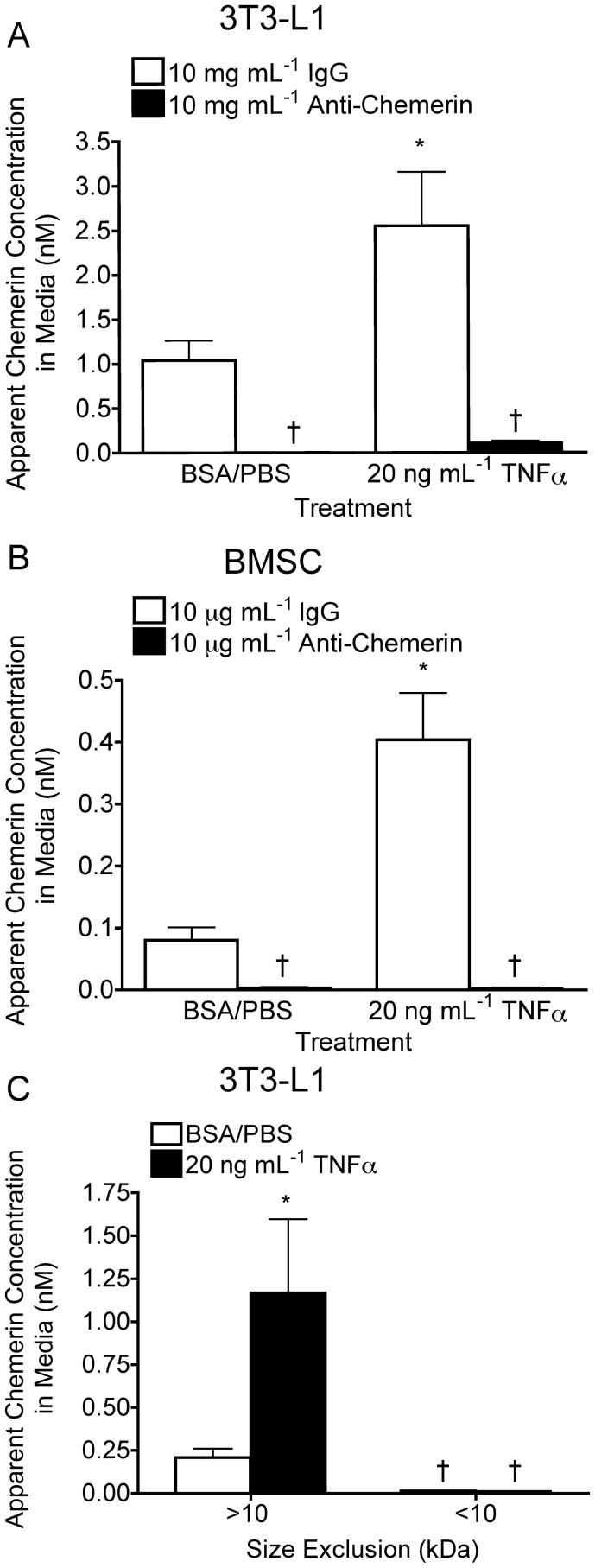
Activation of CMKLR1 by adipocyte media is chemerin-specific. Twenty-four hour conditioned media from 3T3-L1 (**A**, **C**) or BMSC (**B**) adipocytes treated with 20 ng mL^−1^ TNFα or an equivalent volume of the 0.1% BSA/PBS vehicle control were either incubated for 1 hour with 10 µg mL^−1^ of goat anti-mouse chemerin neutralization antibody or 10 µg mL^−1^ IgG control antibody (**A, B**), or separated by size using exclusion column with a 10 kDa molecular weight cutoff (**C**) prior to analysis by CMKLR1 bioassay. All bars represent the mean ± s.e.m. of 3 samples and are representative of 3 independent experiments. *P<0.05 compared to the goat IgG, 0.1% PBS/BSA control (**A, B**) or the 0.1% BSA/PBS control (**C**) and ^†^ P<0.05 compared to the within group goat IgG control (**A, B**) and the respective >10 kDa group (**C**), two-way ANOVA, followed by Bonferroni’s *post hoc* test.

Previous *in vitro* studies have shown CMKLR1 activation can be attributed to full-length chemerin products, small peptide fragments deriving from the chemerin C-terminus and the omega-3 fatty acid derivative resolvin E1 [29], [34], [41]. Therefore, it was necessary to begin our studies by confirming the specificity of CMKLR1 activation by full-length chemerin in adipocyte media under basal and TNFα-treated conditions. To do this, 24 h conditioned serum free medias from 3T3-L1 or BMSC adipocytes were neutralized with an anti-mouse chemerin antibody prior to performing the CMKLR1 bioassay. The anti-mouse chemerin antibody neutralized between 95–99% of the apparent active chemerin concentration in conditioned media obtained from TNFα- or vehicle control-treated 3T3-L1 (Figure 1A) and BMSC (Figure 1B) adipocytes. Following separation of conditioned 3T3-L1 adipocyte media by size exclusion columns, the CMKLR1 bioassay detected high concentrations of active chemerin in the >10 kDa fraction (Figure 1C). In comparison, the apparent chemerin concentration was minimal or undetectable in the <10-kDa fraction. These results confirm that under basal conditions and following treatment with TNFα, activation of CMKLR1 in the bioassay by conditioned adipocyte media is chemerin specific and the active chemerin product(s) generated in the adipocyte media must be greater then 10 kDa.

### Adipocytes Express Immunocyte and Fibrinolytic Associated Enzymes

**Figure 2:**
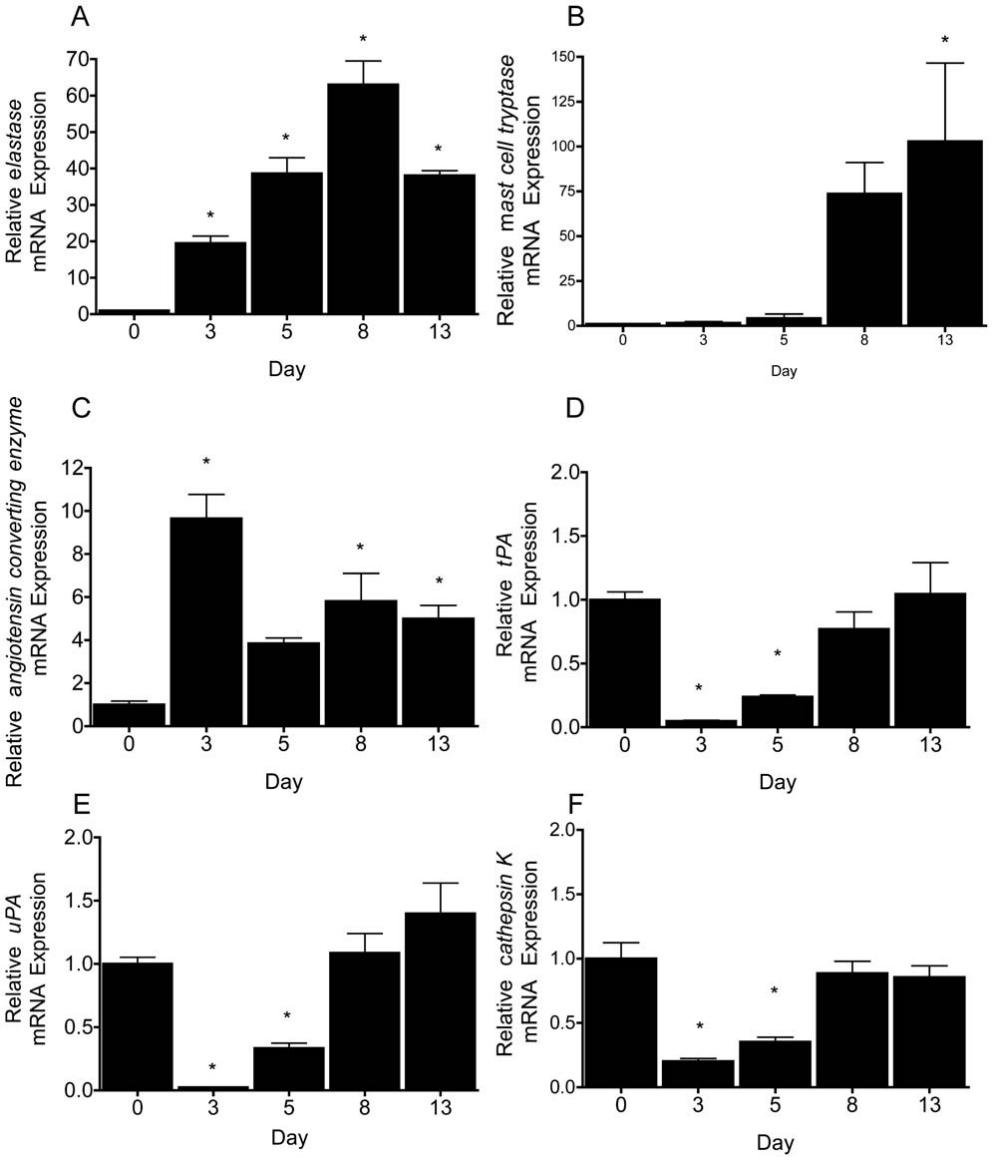
3T3-L1 adipocytes express immunocyte and fibrinolytic associated enzymes. The mRNA expression of *neutrophil elastase* (**A**), *mast cell tryptase* (**B**), *angiotensin converting enzyme* (**C**), *tissue plasminogen activator (tPA)* (**D**), *tissue plasminogen activator urokinase (uPA)* (**E**) and *cathepsin K* (**F**) were analyzed in 3T3-L1 adipocytes throughout differentiation. All bars represent the mean ± s.e.m. of 3 samples and are representative of 3 independent experiments. * P<0.05 compared to the control (D0, preadipocytes), one-way ANOVA, followed by Tukey’s *post hoc* test.

The immunocyte and fibrinolytic associated enzymes neutrophil elastase, mast cell tryptase, tPA, uPA, angiotensin converting enzyme, cathepsin K, carboxypeptidase N and carboxypeptidase B are involved in prochemerin and chemerin proteolysis *in vitro* [8], [27], [28], [29], [30], [32], [33], [36]. *Neutrophil elastase, mast cell tryptase and angiotensin converting* enzyme were minimally expressed in preadipocytes (Figures 2A–C). Progressing from the preadipocyte to fully differentiated adipocyte stages there was a differentiation dependent increase of *neutrophil elastase* (100-fold), *mast cell tryptase* (40-fold) and *angiotensin converting enzyme* (5-fold) (Figures 2A–C). In contrast, *tPA*, *uPA*, and *cathepsin K* (Figures 2D–F) were highly expressed in the in preadipocytes. Compared to preadipocytes, the expression levels of *tPA*, *uPA*, and *cathepsin K* were reduced by 70–90% during the early adipogenic stages (days 3–5), and regained their baseline expression in mature adipocytes (days 8–13). *Carboxypeptidase N* and *B* mRNAs were not detectable in preadipocytes or at the different stages of adipogenesis. Together these results support adipocytes express some but not all the genes that encode for enzymes that activate chemerin. Similar profiles were observed in primary BMSC-derived adipocytes (data not shown).

### General Inhibition of Adipocyte Proteases Increases the Apparent Concentration of Active Chemerin in Adipocyte Media

**Figure 3:**
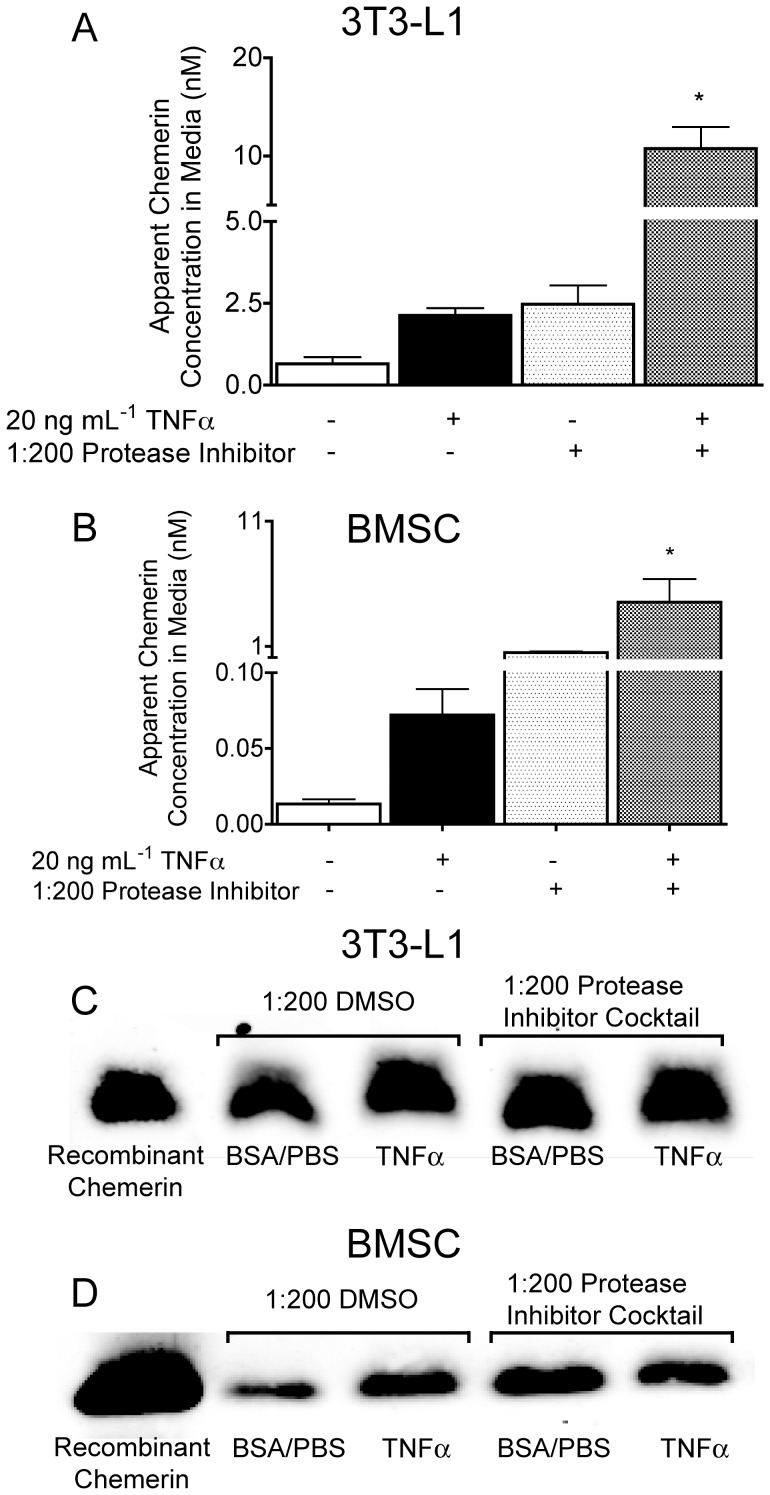
Proteolytic inhibition increases active chemerin concentrations in adipocyte media. 3T3-L1 adipocytes were treated for 24 hours with 20 ng mL^−1^ TNFα or 0.1% BSA/PBS vehicle control in combination with a 1∶200 dilution of a protease inhibitor cocktail (PIC) or its respective vehicle (1∶200 diluted DMSO) prior to analysis by CMKLR1 bioassay (**A, B**) or western blot (**C, D**). All bars represent the mean ± s.e.m. of 3 samples, and are representative of 3 independent experiments. Western blot analysis using an R&D anti-chemerin antibody is representative of 4 samples per group and 3 independent experiments. *P<0.05 compared to the 0.1% BSA/PBS, 1∶200 DMSO vehicle control, two-way ANOVA, followed by Bonferroni’s *post hoc* test.

On the basis that adipocytes express genes that encode for known prochemerin/chemerin proteases, the subsequent goal was to determine whether pan-inhibition of these proteases through the use of a general PIC modifies the adipocyte production of active chemerin. 3T3-L1 or BMSC adipocytes were treated with a vehicle control or TNFα (to induce the production of active chemerin) in the presence and absence of the PIC for 24 h prior to analysis of media samples by the CMKLR1 bioassay. The apparent active chemerin concentration in 3T3-L1 adipocyte media was increased 5-fold by TNFα and PIC treatments alone (Figure 3A). Combined treatment with TNFα and PIC interacted to produce a 20-fold increase in the apparent active chemerin concentration compared to control. Similarly, the BMSC adipocyte media apparent active chemerin concentration was increased 5-fold by TNFα, 30-fold by the PIC and 300-fold by the TNFα and PIC combination treatments (Figure 3B). In contrast, treatment of 3T3-L1 (Figure 3C) or BMSC adipocytes (Figure 3D) with TNFα, the PIC or combined TNFα/PIC minimally (1.25-2 fold) increased the immunodetectable levels of total chemerin in 24 h conditioned adipocyte media compared to the DMSO controls. Overall, these data show that a broad spectrum PIC increases active chemerin concentration in adipocyte media without an equivalent alteration in total chemerin protein.

### Serine and Cysteine Protease Inhibitors Attenuate TNFα-dependent Increases in the Apparent Concentration of Active Chemerin in Adipocyte Media

**Figure 4:**
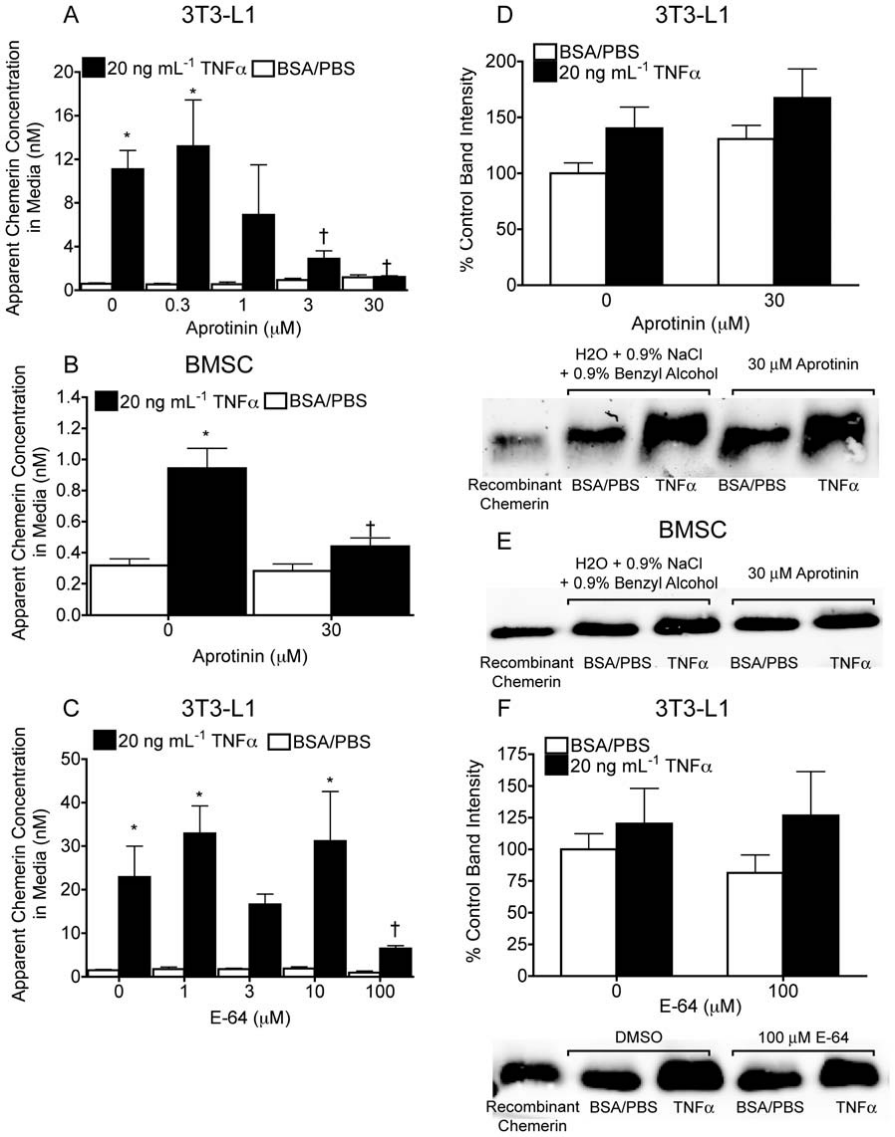
Serine and cysteine protease inhibitors attenuate TNFα-dependent increases in the apparent concentration of active chemerin in adipocyte media. 3T3-L1 or BMSC adipocytes were treated with TNFα or 0.1% BSA/PBS vehicle control in combination with 0–30 µM of the serine protease inhibitor aprotinin or 0–100 µM of the cysteine protease inhibitor E-64 or an equivalent volume of their respective vehicle controls, water or 0.9% NaCl. The effect of these treatments on the apparent concentration of active chemerin in adipocyte media was measured by the CMKLR1 bioassay (A–C). The effect of these treatments on the immunodetectable levels of total chemerin in adipocyte media was measured by western blot and quantified by densitometry (D–F). For densitometry analysis, the dual vehicle treatment (i.e. 0.1% BSA/PBS with H_2_0 or 0.9% NaCl) served as the reference control and was assigned a value of 100%. All bars represent the mean ± s.e.m. of 3 samples and are representative of 3 independent experiments. Western blot using an R&D anti-chemerin antibody is representative of 4 samples per group and 3 independent experiments. * P<0.05 compared to the within group 0.1% BSA/PBS vehicle control, ^†^ P<0.05 compared to the TNFα/vehicle control groups, two-way ANOVA, followed by Bonferroni’s *post hoc* test (A–C).

The PIC experiments provided proof in principle that adipose-derived proteolytic enzymes must be playing a role in determining active chemerin concentration in adipocyte media. On this basis, the subsequent goal was to determine how individual inhibitors that compose the PIC modify the apparent concentration of active chemerin in adipocyte media. We began with serine, and cysteine protease inhibitors aprotinin and E-64 owing to the enzymes’ well-documented role in chemerin activation by immune cells. In TNFα-treated 3T3-L1 adipocytes, aprotinin reduced the apparent media concentration of active chemerin to basal levels in a dose dependent fashion (Figure 4A). The highest dose of aprotinin similarly reduced the apparent concentration of active chemerin in media from TNFα-treated BMSC-derived adipocytes (Figure 4B). Treatment with 100 µM E-64 also reduced the apparent concentration of active chemerin media from TNFα-treated 3T3-L1 adipocytes, but to a lesser extent than aprotinin (Figure 4C). Aprotinin and E-64 had no affect on the basal apparent concentration of active chemerin in adipocyte media of the vehicle-treated control cells. Consistent with the results presented in Figure 3, 20 ng/mL TNFα treatment only produced very small increases (1.25-2-fold) in total immunodetectable chemerin in 24 h conditioned media from 3T3-L1 or BMSC adipocytes compared to the vehicle control (Figure 4D–F). Aprotinin and E-64 did not significantly alter total immunodetectable chemerin in adipocyte media compared to the respective vehicle and TNFα only treatments (Figure 4D–F). Consistent with the lack of *carboxypeptidase N* and *B* expression in adipocytes, the carboxypeptidase inhibitor had no effect on the basal or TNFα-stimulated increase in active chemerin in 3T3-L1 media (data not shown). In summary, these findings show that serine and to a lesser extent cysteine protease inhibitors block the TNFα-mediated increase in media concentrations of active chemerin without altering immunodetectable levels of total chemerin. Neither aprotinin nor E-64 was identified to have any significant effect on the biological activity produced by recombinant chemerin in the CMKLR1 bioassay (Figure S1). Thus, aprotinin and E-64 can be implicated in the reduction of active chemerin formation by adipocytes through inhibition of respective proteases rather than exerting a non-specific effect on the CMKLR1 bioassay.

### Elastase and Tryptase are Increased after Treatment with TNFα

**Figure 5:**
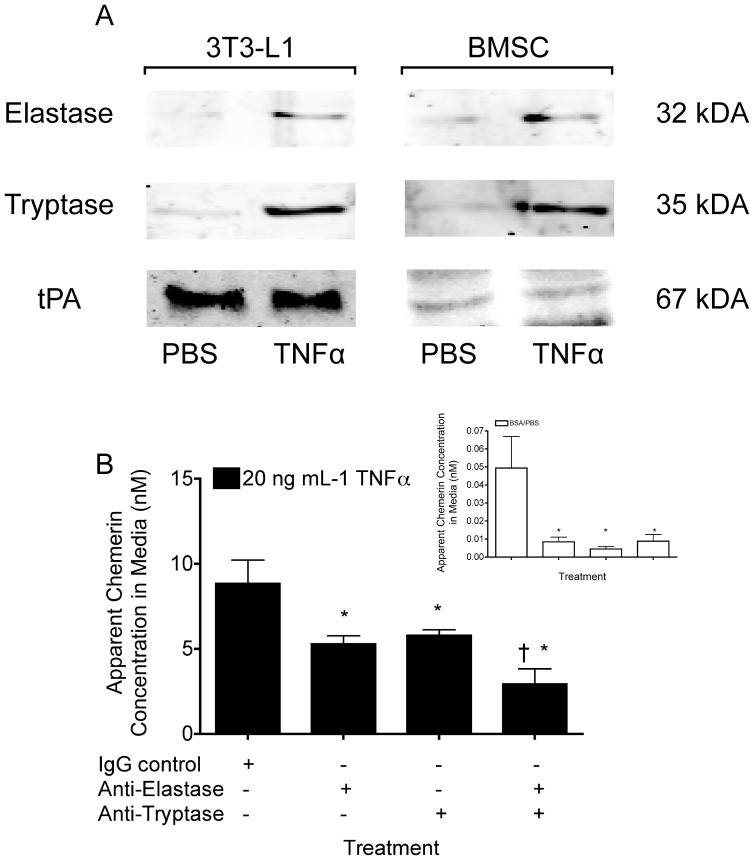
Elastase and tryptase are responsible for the TNFα-mediated increase in the apparent concentration of active chemerin in adipocyte media. The concentrations of elastase, tryptase, and tPA in 24 hour conditioned media from 3T3-L1 and BMSC adipocytes treated with 20 ng mL^−1^ of TNFα or equivalent volume of 0.1% BSA/PBS vehicle control were measured by western blot analysis (A). The apparent concentration of active chemerin in 24 h conditioned media from 3T3-L1 adipocytes treated with 20 ng mL^−1^ of TNFα (B) or equivalent volume of 0.1% BSA/PBS (B, Inset) together with neutralizing antibodies for elastase and tryptase (alone or in combination) or IgG control was measured using the CMKLR1 bioassay. All bars represent the mean ± s.e.m. of 3 samples, and are representative of 2 independent experiments. Western blots are representative of 4 samples per group and 3 independent experiments. * P<0.05 compared to the TNFα/IgG or the 0.1% BSA/PBS/IgG (Inset) treated cells, ^†^ P<0.05 compared to the TNFα+anti-elastase or anti-tryptase treated cells, two-way ANOVA, followed by Bonferroni’s *post hoc* test.

Approximately 90% of the TNFα-mediated increase in apparent active chemerin concentration was inhibited by aprotinin at concentrations ranging from 3–30 µM. These concentrations of aprotinin correspond to known IC_50_ values of tryptase, elastase, tPA and uPA [42], [43]. We next explored whether the expression or secretion profiles of these enzymes were altered in 3T3-L1 and BMSC adipocytes treated with TNFα compared to the PBS control. QPCR analysis at 0, 2, 4 and 8 h after treatment with PBS or TNFα showed little effect of either treatment on the expression profile of these enzymes in 3T3-L1 adipocytes with a slight elevation in *uPA* expression and non-significant depression of *elastase* expression at 8 and 2 hours, respectively (Table S1). Western blot analysis in contrast, identified a 2-fold increase in mast cell tryptase and neutrophil elastase in 24 h conditioned media from both 3T3-L1 and BMSC adipocytes treated with TNFα compared to the PBS control. The amount of tPA in adipocyte media was not affected by TNFα treatment and uPA was not detected in adipocyte media by western blot analysis (Figure 5A).

The elevated levels of elastase and tryptase in 3T3-L1 and BMSC adipocyte media following treatment with TNFα implicated them as probable serine proteases responsible for the heightened apparent active chemerin concentrations. Supporting this, both anti-tryptase and anti-elastase alone inhibited the TNFα-mediated increase in the adipocyte media apparent active chemerin concentration by 40 and 35%, respectively, compared to the IgG control in 3T3-L1 cells (Figure 5B). Combined, the anti-elastase and anti-tryptase treatments had an additive effect neutralizing 67% of the TNFα-associated increase in active chemerin. Under basal conditions anti-tryptase and anti-elastase either separately or together neutralized 85% of the apparent active chemerin concentration. Adipose-derived elastase and tryptase are therefore are important for the formation of active chemerin in the basal state and following TNFα treatment.

### Bestatin Amplifies the TNFα-associated Increase in Active Chemerin in Adipocyte Media

**Figure 6:**
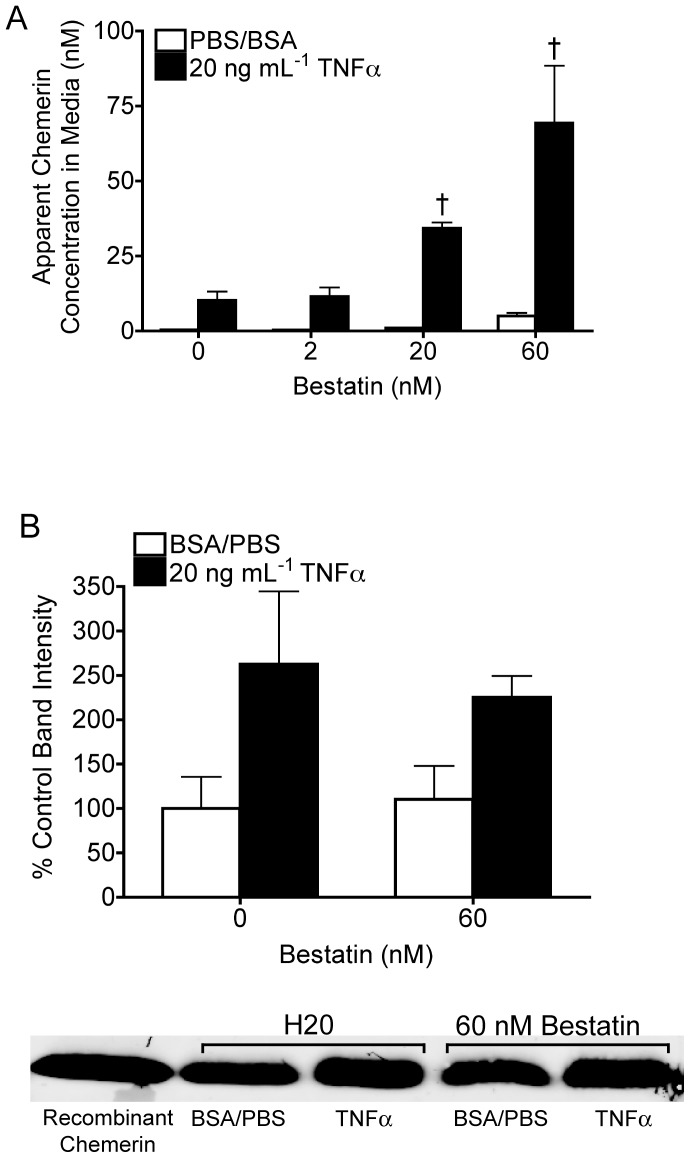
Bestatin heightens the apparent adipocyte media concentration of active chemerin. CMKLR1 bioassay and western blot analysis were used to identify the effect of bestatin, an inhibitor of aminopeptidases, alone and in combination with TNFα or a vehicle control on the apparent (**A**) and total (**B**) media chemerin concentration of 3T3-L1 adipocytes. All bars represent the mean ± s.e.m. of 3 samples and are representative of 3 independent experiments. Western blot using an R&D anti-chemerin antibody is representative of 4 samples per group and 3 independent experiments. ^†^ P<0.05 compared to the TNFα/vehicle control, two-way ANOVA followed by Bonferroni’s *post hoc* test (**A**).

*In vitro* studies have identified that post-translational C-terminal modification by proteases as a critical mechanism in the regulation of chemerin activation. A role for the N-terminus in chemerin functionality has however been largely ignored. Bestatin was consequently investigated for its effect on the apparent concentration of chemerin in 3T3-L1 adipocyte media, as it is both an inhibitor of aminopeptidases, proteases that cleave the N-terminus of proteins and a component of the PIC. Treatment with 3–60 nM bestatin recapitulated the effect of the PIC by further increasing the concentration of active chemerin in 3T3-L1 adipocyte media by 4- and 7-fold compared to the TNFα only treatment (Figure 6A). Similarly, 60 nM bestatin produced a small but non-significant increase in the apparent active chemerin concentration in 3T3-L1 adipocyte media under basal conditions. Bestatin did not increase immunodetectable levels of total chemerin in the media of control cells or following TNFα treatment. Like aprotinin and E-64, 60 nM bestatin had no affect alone on the activity of recombinant mouse chemerin_156_ in the CMKLR1 bioassay (Figure S1). Bestatin is therefore modifying the apparent concentration of active chemerin in adipocyte media rather then non-specifically altering the CMKLR1 bioassay. In summary, these data show that an aminopeptidase inhibitor increases adipocyte production of active chemerin without altering immunodetectable levels of total chemerin.

## Discussion

Chemerin has recently emerged as a regulator of a number of diverse processes including; immune function, cell differentiation and metabolism by signaling through CMKLR1 [2], [3], [44], [45], [46]. In our previously published study we identified that TNFα-treated adipocytes increased their production and secretion of biologically active chemerin [38]. We have also found that the TNFα-associated increase in active chemerin triggers a greater CMKLR1-mediated functional response as measured by the transwell migration of CMKLR1-expressing murine pre-B lymphoma cells (Figure S2). Expanding on those earlier findings, we now report that a neutralizing chemerin antibody prevented CMKLR1 activation by conditioned-adipocyte media. This rules out the possibility that adipocytes are producing non-chemerin ligands of CMKLR1, such as Resolvin E1 [41]. The activation of CMKLR1 was isolated to the adipocyte media fraction containing proteins >10 kDa. This indicates that mouse adipocytes are producing significant quantities of full-length chemerin products, which are between 15-16 kDa in size rather than C-terminal chemerin peptides, which based on their sequences would be less then 3 kDa in size [9], [27], [28], [29], [33], [34], [35], [36], [37], [41], [46], [47], [48].

Mammalian proteases are categorized based on substrate specificity or catalytic mechanisms through which they participate in varied processes including transcription, cell proliferation, differentiation, tissue morphogenesis, tissue remodeling and inflammation by regulating the degradation, localization and activity of proteins [49], [50], [51]. Accordingly, protease inhibition, over-expression and irregular protease function contribute to the pathophysiology of diseases including rheumatoid arthritis and obesity [52]–[57]. Our findings confirmed that mouse adipocytes express a number of immunocyte and fibrinolytic-associated enzymes including *uPA, tPA*, *tryptase, elastase and cathepsin k* [58], [59], [60]. The differentiation-dependent increase in these proteases corresponds well to our previous demonstration of increased active chemerin production by 3T3-L1 adipocytes that occurred between days 3 to 15 after induction of differentiation [2], [3], [44], [45], [46]. Based on the observed expression profiles of these protease genes, we theorized that their production by adipocytes mediates prochemerin activation and therefore the biological functions resulting from chemerin/CMKLR1 signaling. Our initial experiments with a general PIC expanded on this concept supporting the principle that adipocytes are capable of producing proteases that regulate chemerin’s ability to activate CMKLR1. Given the requirement for proteolytic activation of prochemerin prior to CMKLR1 activation, we expected to observe a reduction in chemerin activity following treatment with the general PIC. The PIC had the opposite effect, increasing active chemerin under both basal and TNFα treatment conditions. This result indicated that at the manufacturer’s suggested dilution, the PIC had a net inhibitory effect on the degradation of active chemerin, while prochemerin-activating proteases must have also remained partially active. One notable limitation in using the PIC is that due to its proprietary composition one is unable to determine the concentration of the individual inhibitors that comprise the cocktail. Accordingly, inferences concerning the contributions of individual inhibitors on adipocyte activation of chemerin could not be made. To overcome this limitation we subsequently opted to complete further experiments using individual protease inhibitors.

Serine proteases are well-defined contributors to inflammation [49], [54], [61], [62], [63]. They proteolytically modify chemokines and cytokines increasing both their respective receptor affinities and resulting inflammatory signals [49]. Aprotinin, a serine protease inhibitor, suppressed active chemerin production in TNFα treated 3T3-L1 and BMSC adipocytes. This result identified for the first time that adipocyte-derived serine proteases contribute to enhanced production of active chemerin following TNFα-treatment. Aprotinin inhibits the serine proteases elastase, tryptase, tPA and uPA at the concentrations used. Consequently, it is possible that one or more of these enzymes were involved in prochemerin activation to chemerin by adipocytes [42], [43]. In follow-up experiments, we observed that treatment with TNFα increased adipocyte media concentrations of elastase and tryptase while their combined targeted neutralization recapitulated the aprotinin effect by reducing the concentration of active chemerin in conditioned media. These results support that elastase and tryptase are key mediators of the TNFα-associated increase in apparent active chemerin. The discrepancy between the expression profiles of elastase and tryptase mRNAs and proteins suggested that adipocytes increase the levels of elastase and tryptase following treatment with TNFα through translational or secretory pathways rather than increased gene transcription. This is consistent with enzymatic regulation in other cell types, in which pre-formed proteolytic enzymes are rapidly released as a result of cellular activation [64].

Treating adipocytes with either aprotinin or elastase and tryptase antibodies could not, however, completely inhibit basal or TNFα-associated chemerin activity. Thus, serine proteases are major but not exclusive regulators of chemerin activation. The reduction of apparent active chemerin in adipocyte media following co-treatment with E-64 and TNFα suggests that cysteine proteases mediate a minor component of chemerin activation. While tPA was detected in adipocyte media, its concentration did not increase with TNFα treatment, and uPA was undetectable suggesting that these serine proteases are less likely to be involved in TNFα-associated elevations in adipocyte-prochemerin processing. Furthermore, the lack of both *carboxypeptidase B* and *N* mRNA expression and functional effects of the carboxypeptidase inhibitor indicate that carboxypeptidase B and N are not involved in adipocyte-prochemerin processing.

**Figure 7:**
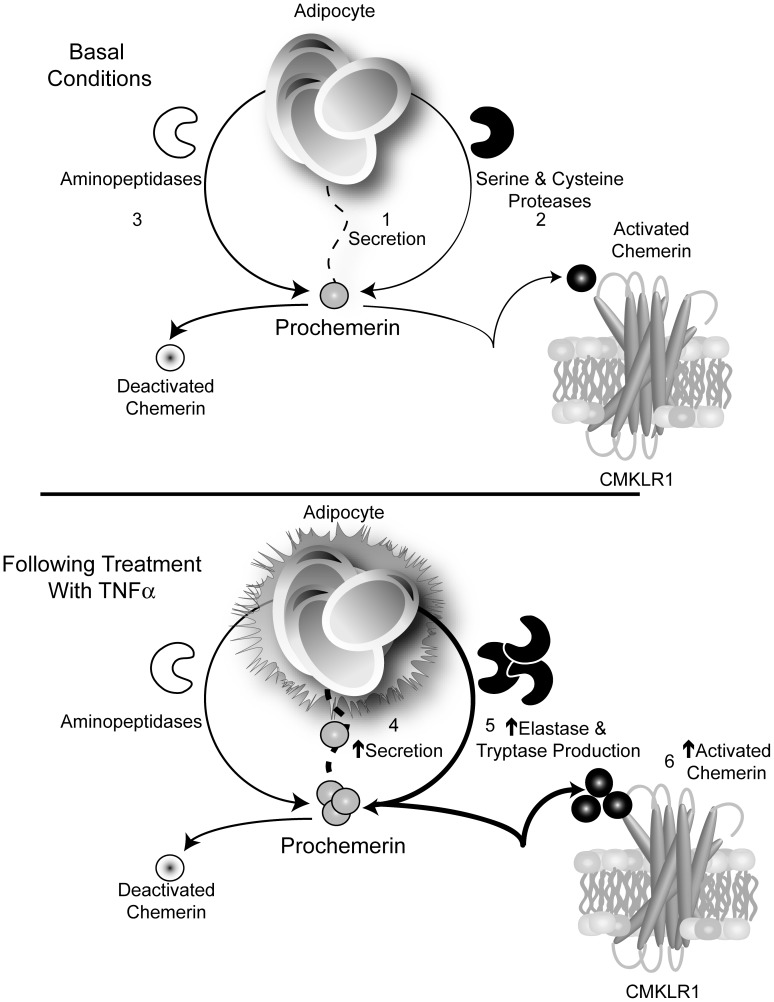
Working model of adipocyte-derived proteolytic control of active chemerin under basal conditions and following treatment with TNFα. Our findings together support a model of adipocyte-derived proteolytic control of chemerin activity. Under basal conditions the activity of adipocyte-secreted chemerin (1) at CMKLR1 is determined by a precise balance between activation by serine and cysteine protease (2) and deactivation by aminopeptidases (3). Following treatment with TNFα, elevated secretion of chemerin (4) and production of elastase and tryptase (5) alter this balance resulting in increased concentration of a chemerin product(s) with high activity towards CMKLR1 (6).

Elastase and tryptase are generated from their respective inactive zymogens, proelastase and protryptase, by proteolytic processing [65], [66], [67]. Thus, adipocytes must also process protryptase and proelastase to their active forms. However, the mechanisms that mediate elastase and tryptase activation by adipocytes remain elusive. A possibility we considered was dipeptidylpeptidase IV (DPP-IV) as DPP-IV may be linked to proelastase maturation through proteolytic cascades; DPP-IV has been recently identified as an adipokine with heightened expression in obese adipose tissue and DPP-IV inhibitors are used for treatment of diabetes [59], [68], [69], [70]. The DPP-IV inhibitor KR-62436 at concentrations at or above its IC_50_ value for DDP-IV had no affect on basal or TNFα-associated increases in active chemerin in 3T3-L1 adipocyte media (data not shown). A second DPP-IV inhibitor sitagliptin at 20 nM (a concentration equal to its IC_50_ for DPP-IV) produced a minor inhibition (30%) of the TNFα-mediated increase in active chemerin but was without effect at 100 nM. Thus, DPP-IV appears to have minimal involvement in chemerin activation by 3T3-L1 adipocytes. Other proteolytic mechanisms that should be considered in future studies are DPP-1 and cathepsin B and L, which are known activators of proelastase and protryptase in other cell types [66], [67].

Studies investigating chemerin functionality have exclusively focused on processing of the prochemerin C-terminus. The importance of the chemerin C-terminus for CMKLR1 activation is supported by the capacity of synthetic peptides corresponding to the final 9 to 19 amino acids of human chemerin_157_ to bind and activate CMKLR1 [29], [40], [71]. However, their inability to bind with the same potency as full-length chemerin_157_ indicates N-terminal regions are required for optimal ligand-receptor interactions. To explore the possibility of N-terminal processing of chemerin we focused on the inhibition of aminopeptidases, which are distributed ubiquitously throughout tissue and body fluids and are involved in protein inactivation via proteolytic cleavage of N-terminal amino acids of designated proteins including IL-8 and enkephalin [72], [73], [74], [75]. The observation that bestatin significantly enhanced the TNFα-mediated increase in apparent active chemerin concentration in adipocyte media suggested that aminopeptidases are involved in chemerin inactivation through proteolytic modification of the protein’s N-terminus. Notwithstanding, further studies to identify the specific aminopeptidases involved and the manner in which they regulate active chemerin are needed, but are well beyond the scope of the current manuscript.

Based on our overall results we have developed an initial working model for white adipocyte chemerin processing (Figure 7). It is proposed that adipocytes secrete serine and cysteine proteases that activate chemerin and aminopeptidases that terminate chemerin bioactivity. A balance between these activation and termination mechanisms produce a low level of active chemerin in the basal state as measured by CMKLR1 activation (Figure 7, top). Treating adipocytes with TNFα increases serine protease activation of chemerin more so than it terminates chemerin activity via increased aminopeptidases resulting in a net increase in active chemerin with potent agonist activity at CMKLR1 (Figure 7, bottom). Conservation of the majority of our results in two complementary adipocyte models supports the importance of these adipocyte-derived proteases in controlling chemerin activation.

Many recent human studies have found positive associations between chemerin and markers of obesity, inflammation and measures of metabolic syndrome indicating that chemerin concentrations are modifiable in obesity and may contribute to human diseases associated with obesity or inflammation [10], [11], [12], [26], [76]. The ELISAs and western blot protocols used in those studies, however, provided a measurement of total chemerin with no method of differentiating between pro-, active and inactive chemerin. Inferring a role for chemerin in the pathophysiology of diseases has therefore rested exclusively on an assumption of parity between the concentrations of total circulating chemerin equating to its activity at CMKLR1. Our finding that TNFα produced a 10-fold increase in the apparent active concentrations of chemerin in adipocyte media while only increasing total chemerin by 1.25-2-fold indicates that it is inaccurate to assume such parity exists. This lack of parity is further emphasized by the protease inhibitor treatments, which increased (PIC and bestatin) or supressed (aprotinin, E64 and elastase and tryptase neutralization) the TNFα-mediated active chemerin output without similarly altering immunodetectable chemerin. The identified disparity between immunodetectable chemerin concentration and its associated activity at CMKLR1 also supports that protease inhibitors are interfering with mechanisms of posttranslational processing of prochemerin/chemerin that are normally activated by TNFα treatment. Possible mechanisms may include single C-terminal or N-terminal cleavages and/or modifications to protein secondary and tertiary structures that would enhance or diminish chemerin’s ability to activate or even antagonize CMKLR1 [9], [28], [29], [30], [32], [33], [36], [77]. These molecular changes would not be resolved by western blot analysis or traditional chemerin ELISAs given the similar molecular weights of the chemerin products, the non-specific nature of the antibodies and the denaturing conditions of the western assay. Taken together, the disparity between active and immunodetectable chemerin warrants that caution should be taken when interpreting data from traditional chemerin ELISAs or western blots as they may not accurately predict active chemerin concentrations especially in situations where an inflammatory response is present, which includes obesity.

In summary, our results indicated that adipocytes could be a key site for extravascular proteolytic processing of prochemerin. Other evidence supporting a role for extravascular proteolytic factors in the production of active chemerin comes from a recent human chemerin study by Zhao et al. Using selective human prochemerin and chemerin ELISAs, they detected an increased fraction of active chemerin_157+158_ in the synovial fluid of rheumatoid arthritis and osteoarthritis patients compared to healthy human plasma [71]. This result indicated that there is a significant shift towards proteolytic production of active forms of chemerin *in vivo* in the synovial fluid in humans with inflammatory joint diseases. Thus, based on our results it should be a priority for future studies to determine if adipose tissue inflammation that occurs with obesity imposes a similar situation in which elevated adipocyte proteolytic mechanisms lead to increased local or circulating concentrations of active chemerin. To overcome the limitation of traditional chemerin ELISAs it is imperative these studies include measures of both total chemerin and active chemerin, as in our study, or alternatively, antibodies that recognize and quantify specific chemerin products. In this way needed insight concerning the regulation of chemerin activity and the relationship between elevated total chemerin levels in biological fluids (e.g. plasma and serum) and obesity or inflammation will be elucidated.

## Supporting Information

Figure S1 **Aprotinin, E-64 and bestatin do not non-specifically affect the CMKLR1 bioassay.** To rule out non-specific effects of aprotinin, E-64 or bestatin on the assay itself, the luciferase/β-galactosidase activity of varying recombinant chemerin standards combined with 30 µM aprotinin (**A**), 100 µM E-64 (**B**), 60 nM bestatin (**C**) or their respective controls were analyzed by CMKLR1 bioassay. All bars represent the mean ± s.e.m. of 3 samples and are representative of 2 independent experiments.
(TIF)

Figure S2 **TNFα increases adipocyte-derived chemerin recruitment of human CMKLR1-expressing pre-B lymphoma cells.** Twenty-four hour serum free conditioned media from 3T3-L1 adipocytes treated with 0.1% BSA/PBS or 1 ng mL^−1^ TNFα were tested for the ability to stimulate migration of human CMKLR1-L1.2 or empty vector (pcDNA3-L1.2) expressing murine pre-B lymphoma cells through a 5 µm transwell insert as previously described in detail [2]. All bars represent the mean ± s.e.m. of 3 samples. * P<0.05 compared to L1.2-pcDNA cells, † p<0.05 compared to vehicle control, two-way ANOVA, followed by Bonferroni *post hoc* test.
(TIF)

Table S1 **Relative expression of immune and fibrinolytic enzymes in 3T3-L1 adipocytes following TNFα treatment.** 3T3-L1 adipocytes were treated with 20 ng mL**^−^** ^1^ TNFα or an equivalent volume of 0.1% BSA/PBS vehicle after which they were harvested at 0, 2, 4 and 8 hours after treatment for gene expression analysis. For determination of relative gene expression by QPCR, the 0 time point served as the reference (expression = 1) to which all other sample were compared. Each value is the mean ± s.e.m. of 3 samples. P<0.05, significantly different compared to the control, Two-way ANOVA followed by Bonferroni’s *post-hoc* test.
(DOC)
