# Supplementary figures and images for: Elastase and Tryptase Govern TNFα-Mediated Production of Active Chemerin by Adipocytes
Source: PLoS One. 2012 Dec 5;7(12):e51072. doi: 10.1371/journal.pone.0051072 (PMC3515524; doi:10.1371/journal.pone.0051072)

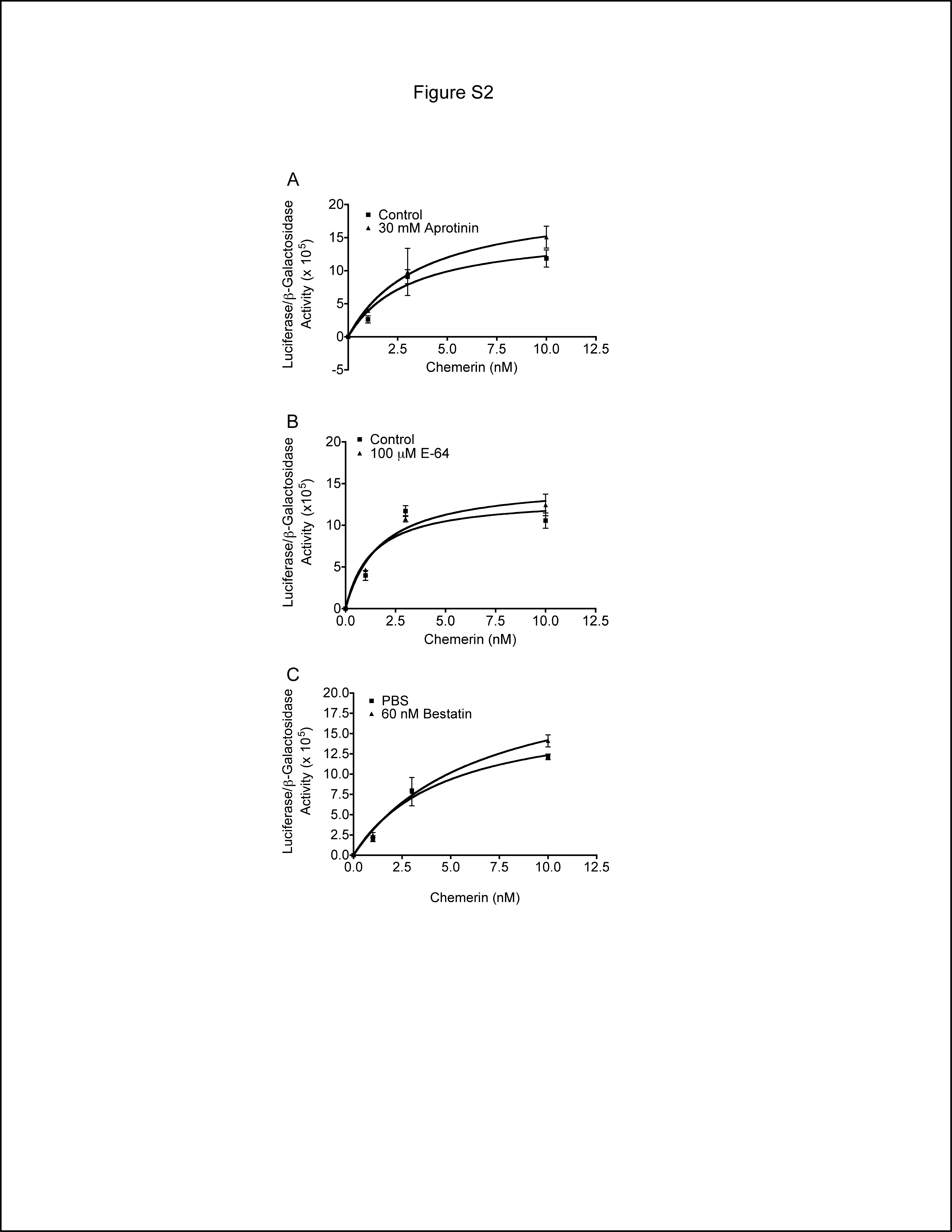

Supplement: Figure S1 — Aprotinin, E-64 and bestatin do not non-specifically affect the CMKLR1 bioassay. To rule out non-specific effects of aprotinin, E-64 or bestatin on the assay itself, the luciferase/β-galactosidase activity of varying recombinant chemerin standards combined with 30 µM aprotinin (A), 100 µM E-64 (B), 60 nM bestatin (C) or their respective controls were analyzed by CMKLR1 bioassay. All bars represent the mean ± s.e.m. of 3 samples and are representative of 2 independent experiments. (TIF) [file pone.0051072.s001.tif]

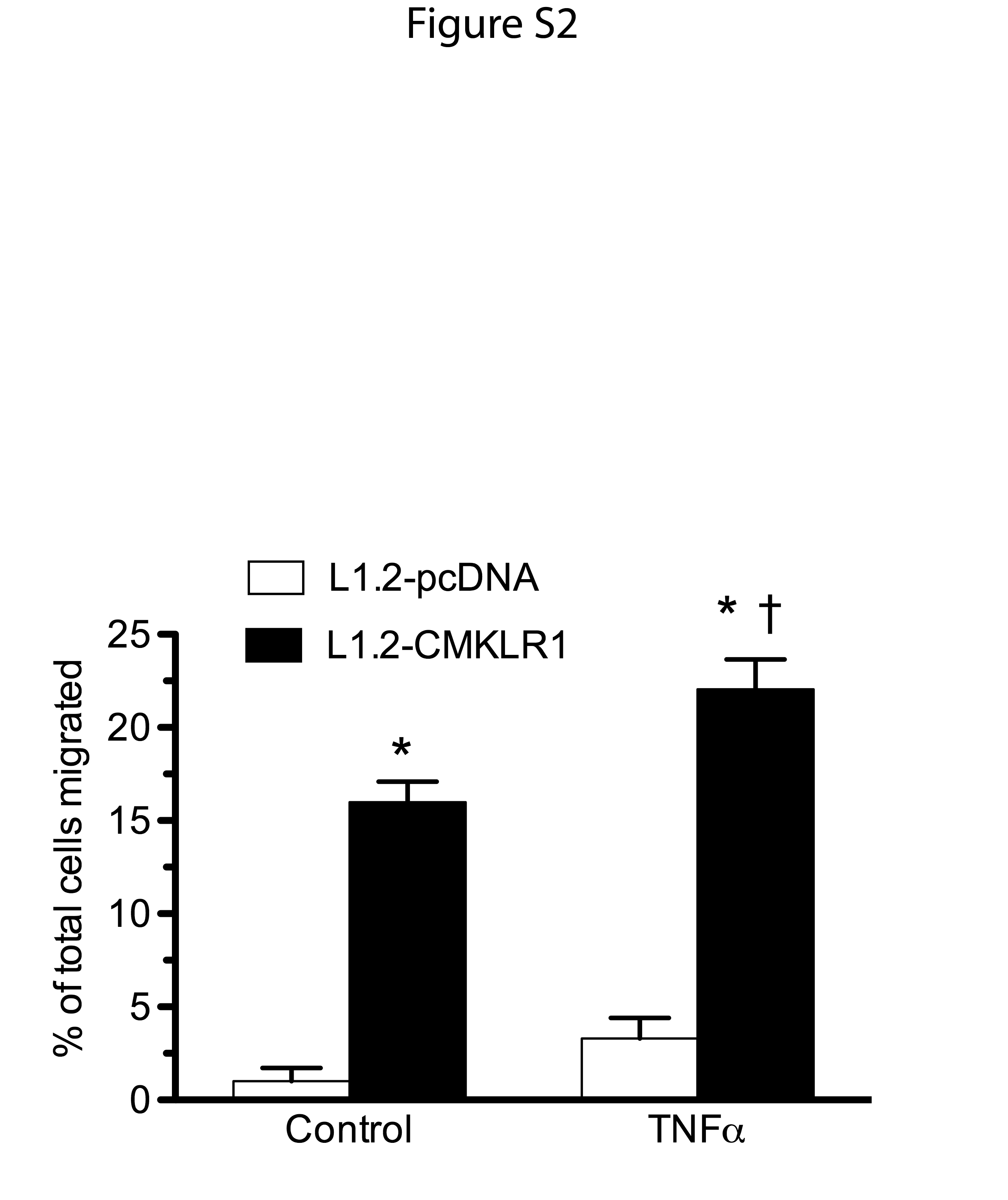

Supplement: Figure S2 — TNFα increases adipocyte-derived chemerin recruitment of human CMKLR1-expressing pre-B lymphoma cells. Twenty-four hour serum free conditioned media from 3T3-L1 adipocytes treated with 0.1% BSA/PBS or 1 ng mL−1 TNFα were tested for the ability to stimulate migration of human CMKLR1-L1.2 or empty vector (pcDNA3-L1.2) expressing murine pre-B lymphoma cells through a 5 µm transwell insert as previously described in detail [2]. All bars represent the mean ± s.e.m. of 3 samples. * P<0.05 compared to L1.2-pcDNA cells, † p<0.05 compared to vehicle control, two-way ANOVA, followed by Bonferroni post hoc test. (TIF) [file pone.0051072.s002.tif]
