# Supplementary material for: Elastase and Tryptase Govern TNFα-Mediated Production of Active Chemerin by Adipocytes
Source: PLoS One. 2012 Dec 5;7(12):e51072. doi: 10.1371/journal.pone.0051072 (PMC3515524; doi:10.1371/journal.pone.0051072)
Supplement: Table S1 — Relative expression of immune and fibrinolytic enzymes in 3T3-L1 adipocytes following TNFα treatment. 3T3-L1 adipocytes were treated with 20 ng mL− 1 TNFα or an equivalent volume of 0.1% BSA/PBS vehicle after which they were harvested at 0, 2, 4 and 8 hours after treatment for gene expression analysis. For determination of relative gene expression by QPCR, the 0 time point served as the reference (expression = 1) to which all other sample were compared. Each value is the mean ± s.e.m. of 3 samples. P<0.05, significantly different compared to the control, Two-way ANOVA followed by Bonferroni’s post-hoc test. (DOC) [file pone.0051072.s003.doc]

**Table S1:** Relative expression of immune and fibrinolytic enzymes in 3T3-L1 adipocytes following TNFα treatment

| **Gene** | **Time following Treatment (hrs)** | **Treatment** | | | **Statistical** |
| --- | --- | --- | --- | --- | --- |
|  |  | **0.1 % BSA/PBS** | **20 ng mL^-1^ TNFα** | | **P-Value** |
|  |  | **Mean ± S.E.M.** | | |  |
| ***Neutrophil Elastase*** | **0** | 1.00 (0.24) | | 1.00 (0.13) | P > 0.05 |
|  | **2** | 2.12 (0.82) | | 1.02 (0.13) | P > 0.05 |
|  | **4** | 1.76 (0.15) | | 0.80 (0.3) | P > 0.05 |
|  | **8** | 0.94 (0.14) | | 0.19 (0.06) | P > 0.05 |
| ***Mast Cell Tryptase*** | **0** | 1.00 (0.14) | | 1.18 (0.83) | P > 0.05 |
|  | **2** | 1.43 (0.11) | | 2.10 (0.37) | P > 0.05 |
|  | **4** | 1.89 (0.47) | | 1.76 (0.53) | P > 0.05 |
|  | **8** | 1.43 (0.19) | | 1.46 (0.15) | P > 0.05 |
| ***uPA*** | **0** | 1.00 (0.25) | | 0.75 (0.32) | P > 0.05 |
|  | **2** | 0.78 (0.13) | | 2.95 (0.49) | **P <0.05** |
|  | **4** | 0.88 (0.36) | | 1.10 (0.2) | P > 0.05 |
|  | **8** | 0.75 (0.15) | | 0.48 (0.12) | P > 0.05 |
| ***tPA*** | **0** | 1.00 (0.07) | | 1.01 (0.05) | P > 0.05 |
|  | **2** | 1.27 (0.44) | | 1.10 (0.12) | P > 0.05 |
|  | **4** | 0.85 (0.05) | | 0.95 (0.16) | P > 0.05 |
|  | **8** | 0.58 (0.09) | | 1.12 (0.16) | P > 0.05 |
